# Supplementary material for: Molecular and structural basis of anti-DNA antibody specificity for pyrrolated proteins
Source: Commun Biol. 2024 Feb 3;7:149. doi: 10.1038/s42003-024-05851-0 (PMC10838295; doi:10.1038/s42003-024-05851-0)
Supplement: Supplementary file 2 — Supplementary Information [file 42003_2024_5851_MOESM2_ESM.pdf]

Supplementary Information

Molecular and structural basis of anti-DNA antibody specificity for pyrrolated proteins

Yusuke Anan, Masanori Itakura, Tatsuya Shimoda, Kosuke Yamaguchi, Peng Lu, Koji Nagata, Jinhua Dong, Hiroshi Ueda, and Koji Uchida

Supplementary Tables

Supplementary Table 1. Primers used for the construction of scFv library.

Primer name	Sequence
MHV.BACK 1	5'-CTCAGCCGGCCATGGCAGATGTGAAGCTTCAGGAGTC-3'
MHV.BACK 2	5'-CTCAGCCGGCCATGGCACAGGTGCAGCTGAAGGAGTC-3'
MHV.BACK 3	5'-CTCAGCCGGCCATGGCACAGGTGCAGCTGAAGCAGTC-3'
MHV.BACK 4	5'-CTCAGCCGGCCATGGCACAGGTTACTCTGAAAGAGTC-3'
MHV.BACK 5	5'-CTCAGCCGGCCATGGCAGAGGTCCAGCTGCAACAATCT-3'
MHV.BACK 6	5'-CTCAGCCGGCCATGGCAGAGGTCCAGCTGCAGCAGTC-3'
MHV.BACK 7	5'-CTCAGCCGGCCATGGCACAGGTCCAACTGCAGCAGCCT-3'
MHV.BACK 8	5'-CTCAGCCGGCCATGGCAGAGGTGAAGCTGGTGGAGTC-3'
MHV.BACK 9	5'-CTCAGCCGGCCATGGCAGAGGTGAAGCTGGTGGAATC-3'
MHV.BACK 10	5'-CTCAGCCGGCCATGGCAGATGTGAACTTGGAAGTGTC-3'
MHV.FOR 1	5'-CACCTTCTTCAAGCTTTGCAGAGACAGTGACCAGAGT-3'
MHV.FOR 2	5'-CACCTTCTTCAAGCTTTGAGGAGACTGTGAGAGTGGT-3'
MHV.FOR 3	5'-CACCTTCTTCAAGCTTTGAGGAGACGGTGACTGAGGT-3'
MHV.FOR 4	5'-CACCTTCTTCAAGCTTTGAGGAGACGGTGACCGTGGT-3'
MKV.BACK 1	5'-TTCAGAAGCACGCGTAGATGTTTTGATGACCCAAACT-3'
MKV.BACK 2	5'-TTCAGAAGCACGCGTAGATATTGTGATGACGCAGGCT-3'
MKV.BACK 3	5'-TTCAGAAGCACGCGTAGATATTGTGATAACCCAG-3'
MKV.BACK 4	5'-TTCAGAAGCACGCGTAGACATTGTGCTGACCCAATCT-3'
MKV.BACK 5	5'-TTCAGAAGCACGCGTAGACATTGTGATGACCCAGTCT-3'
MKV.BACK 6	5'-TTCAGAAGCACGCGTAGATATTGTGCTAACTCAGTCT-3'
MKV.BACK 7	5'-TTCAGAAGCACGCGTAGATATCCAGATGACACAGACT-3'
MKV.BACK 8	5'-TTCAGAAGCACGCGTAGACATCCAGCTGACTCAGTCT-3'
MKV.BACK 9	5'-TTCAGAAGCACGCGTACAAATTGTTCTCACCCAGTCT-3'
MKV.FOR 1	5'-GGATCCAGCGGCCGCCCGTTTCAGCTCCAGCTTG-3'
MKV.FOR 2	5'-GGATCCAGCGGCCGCCCGTTTTATTTCCAGCTTGGT-3'
MKV.FOR 3	5'-GGATCCAGCGGCCGCCCGTTTTATTTCCAACTTTG-3'
MKC5.FOR	5'-GGATCCAGCGGCCGCGGATACAGTTGGTGCAGCATC-3'
MLV.BACK	5'-TTCAGAAGCACGCGTACAGGCTGTTGTGACTCAGGAA-3'
MLV.FOR	5'-GGATCCAGCGGCCGCCTTGGGCTGACCTAGGACAGT-3'

Supplementary Table 2. Primers used for the synthesis of SARAH domain genes.

Primer name	Sequence
SARAH_1	5'-GGCAGCGATTACGAATTTCTGAAAAGCTGGA CCGTGGAAGATCTGCAGAAACGCCTGCTGGC-3'
SARAH_2	5'-CTGATATTTCTGGCGAATTTCTTCAATTTCCTGTTC CATCATCGGATCCAGCGCCAGCAGGCGTTTCTGC-3'
SARAH_3_VH	5'-GGAAATTGAAGAAATTCGCCAGAAATACCAGAGCA AACGCCAGCCGATTCTGGATGCGATTGAAGCGAAA-3'
SARAH_3_VL	5'-GGAAATTGAAGAAATTCGCCAGAAATATCAGTGCAA ACGTCAGCCGATTCTGGATGCGATTGAAGCGAAA-3'
SARAH_4	5'-TTTCGCTTCAATCGCATCCAGAATC-3'

Supplementary Figures

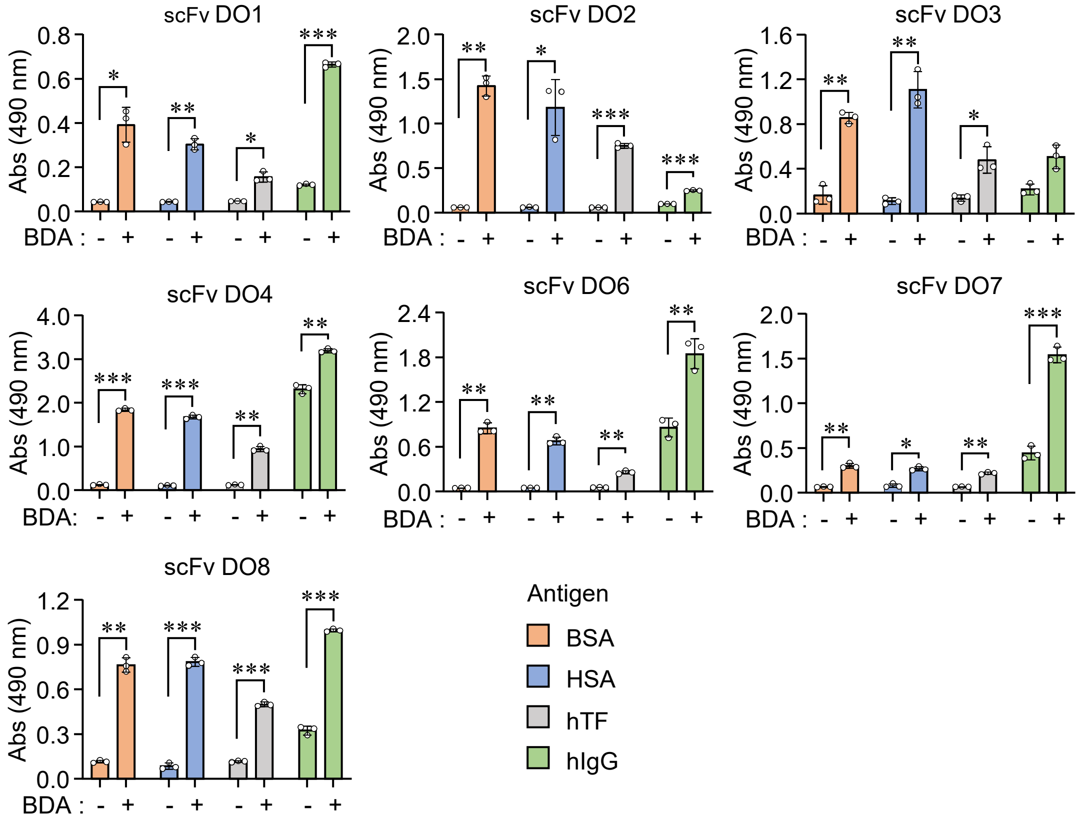

Supplementary Fig. 1. Binding of anti-DNA scFvs to native or pyrrolated proteins. Pyrrolated proteins were prepared by incubating proteins (BSA, HSA, hTF, and hIgG) with BDA. Seven anti-DNA scFvs DO1-DO8, except for scFv DO5, were used for this experimen. Data are mean ± S.D. of triplicate samples (representative of three independent experiments). Student's t test (two-sided), *, $p < 0.05$; **, $p < 0.01$; ***, $p < 0.001$.

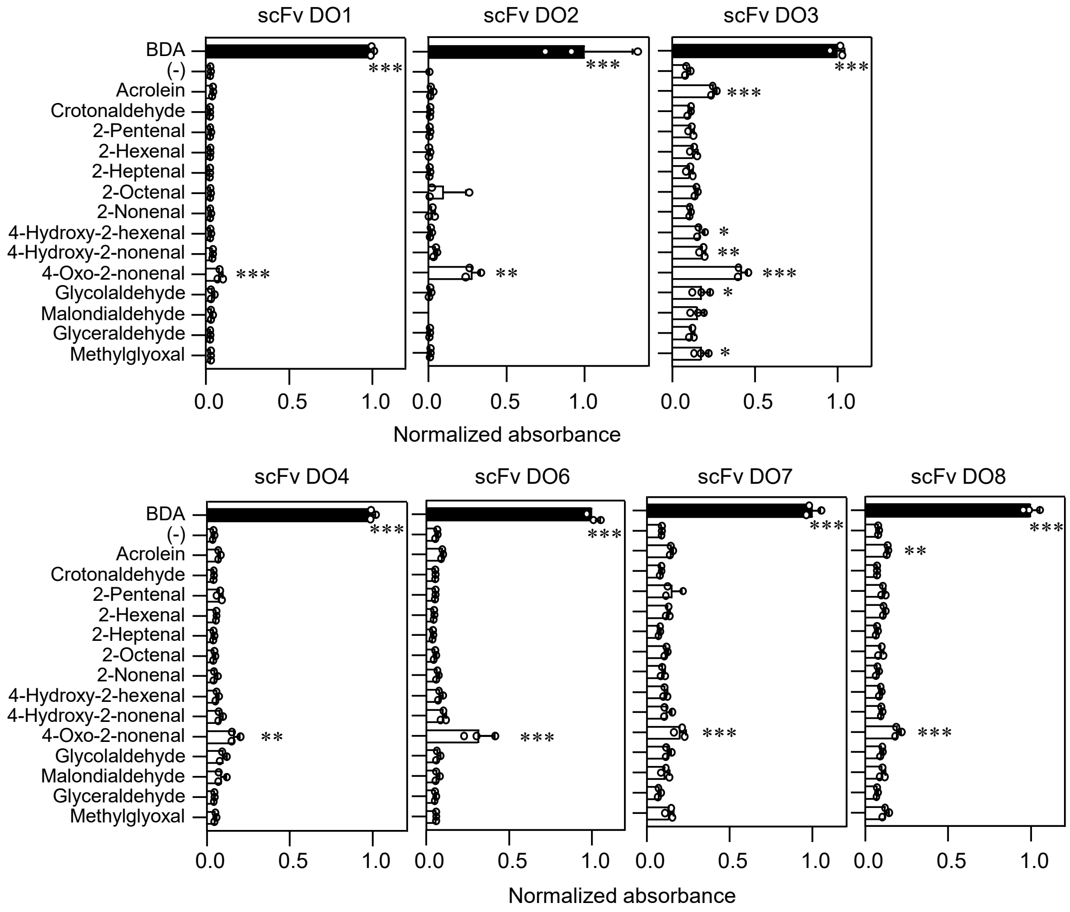

Supplementary Fig. 2. Binding of anti-DNA scFvs DO1-DO8 to aldehyde-modified proteins. Aldehyde-modified proteins were prepared by incubating BSA with aldehydes. Seven anti-DNA scFvs DO1-DO8, except for scFv DO5, were used for this experiment. Antigens (1 μg/well) immobilized on ELISA plate were incubated with scFv-His_6. The absorbance values are normalized by that of pyrBSA (described as BDA). Data are mean ± S.D. of triplicate samples (representative of three independent experiments). Dunnett's test (two-sided), relative to BSA, *, $p < 0.05$; **, $p < 0.01$; ***, $p < 0.001$.

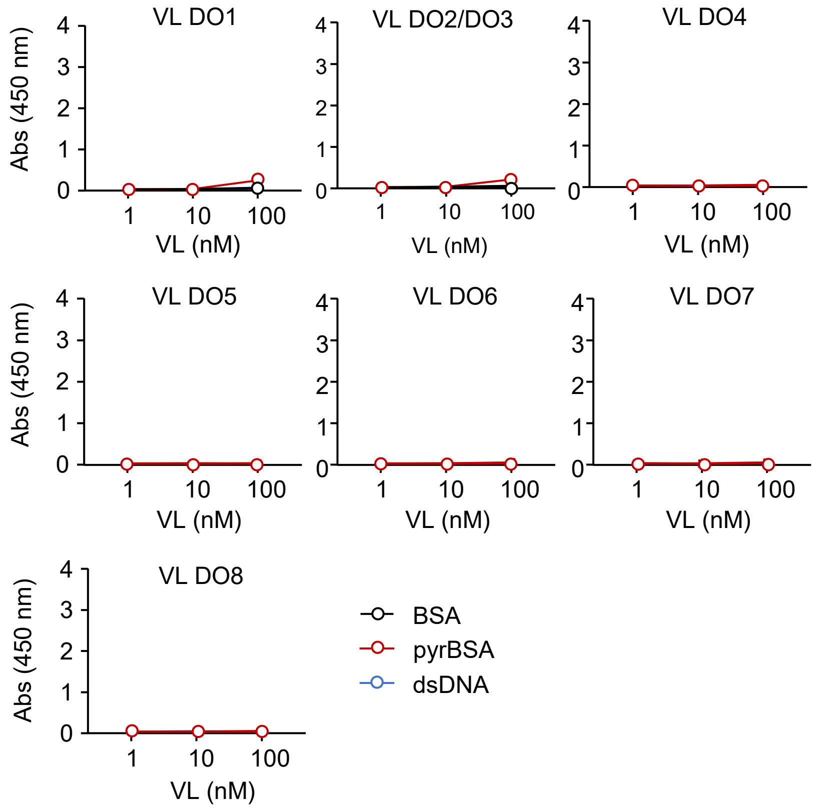

Supplementary Fig. 3. Binding of the VL domain to DNA and pyrP. Binding of VL-His$_6$ to antigens (BSA, pyrBSA, and DNA) was evaluated by ELISA. Antigens (1 µg/well) immobilized on ELISA plate were incubated with VL-His$_6$ (1, 10, and 100 nM each). Data are mean ± S.D. of triplicate samples (representative of three independent experiments).

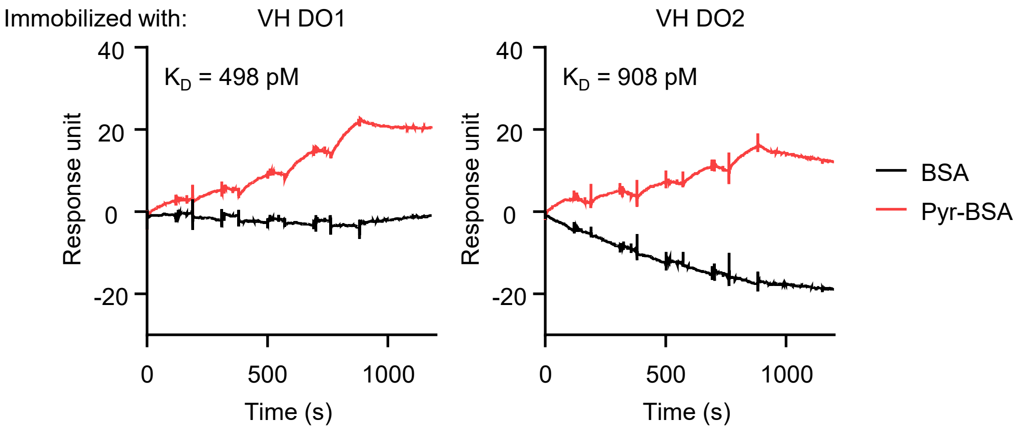

Supplementary Fig 4. Surface plasmon resonance measurements. The interactions of BSA or pyrrolated BSA with VH Abs (DO1 and DO2) immobilized by amine coupling in the lanes of a BIAcore sensor chip NTA were monitored by the single-cycle kinetics method. The surface plasmon resonance assays were performed using a Biacore T100 instrument (GE Healthcare). The VH His-tag were immobilized on a sensor chip NTA (GE Healthcare) at a density of 1000 response units (RU). The interaction between the immobilized VH Abs and pyrrolated BSA (6.25～100 ng/ml) was examined at 25°C with a flow rate of 30 ml/min by a single-cycle kinetics analysis program. HBS-P (10 mM HEPES-NaOH (pH 7.4), 150 nM NaCl, 0.05% Tween 20) was used as the running buffer. The response curves obtained from injecting buffer only and from the control flow cell (without immobilized VH Abs) were subtracted from the VH Abs-immobilized cell to correct for any nonspecific binding. BIAevaluation software (version 4.1) was used to perform the kinetic analysis.

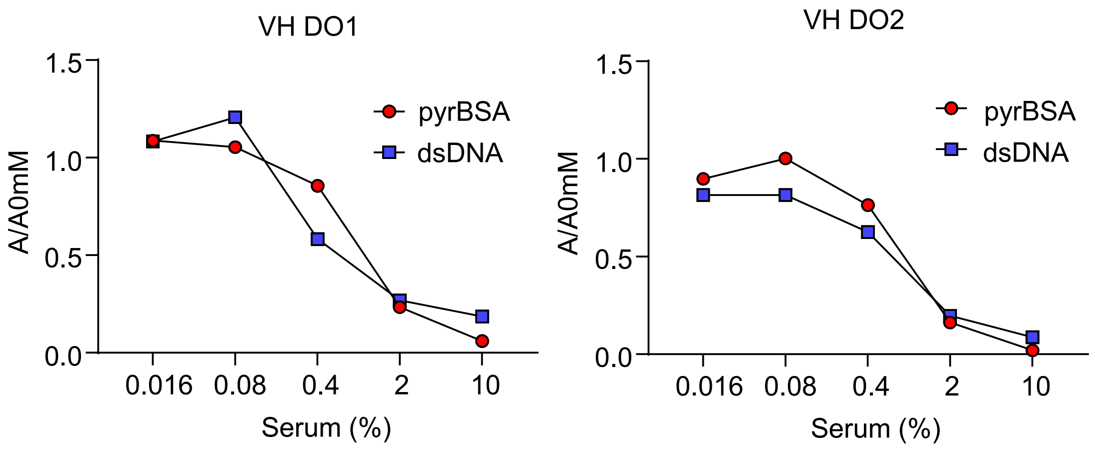

Supplementary Fig. 5. Effect of serum on binding of VH DO1 and DO2 Abs to pyrBSA or dsDNA. pyrBSA or dsDNA (10 μg/ml) immobilized on ELISA plate was incubated with VH Abs in the presence of mouse serum (0.016, 0.08, 0.4, , 2, or 10%).

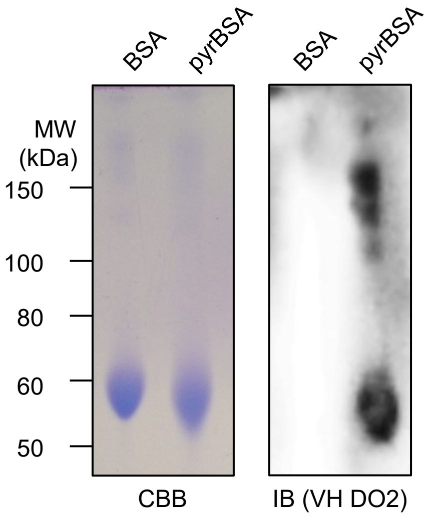

Supplementary Fig. 6. Immunoblot analysis of pyrBSA using VH DO2. BSA and pyrBSA were separated on a 10% SDS polyacrylamide gel, followed by staining with Coomassie brilliant blue (CBB) (*left*) or blotting and detection with VH DO2 (*right*).

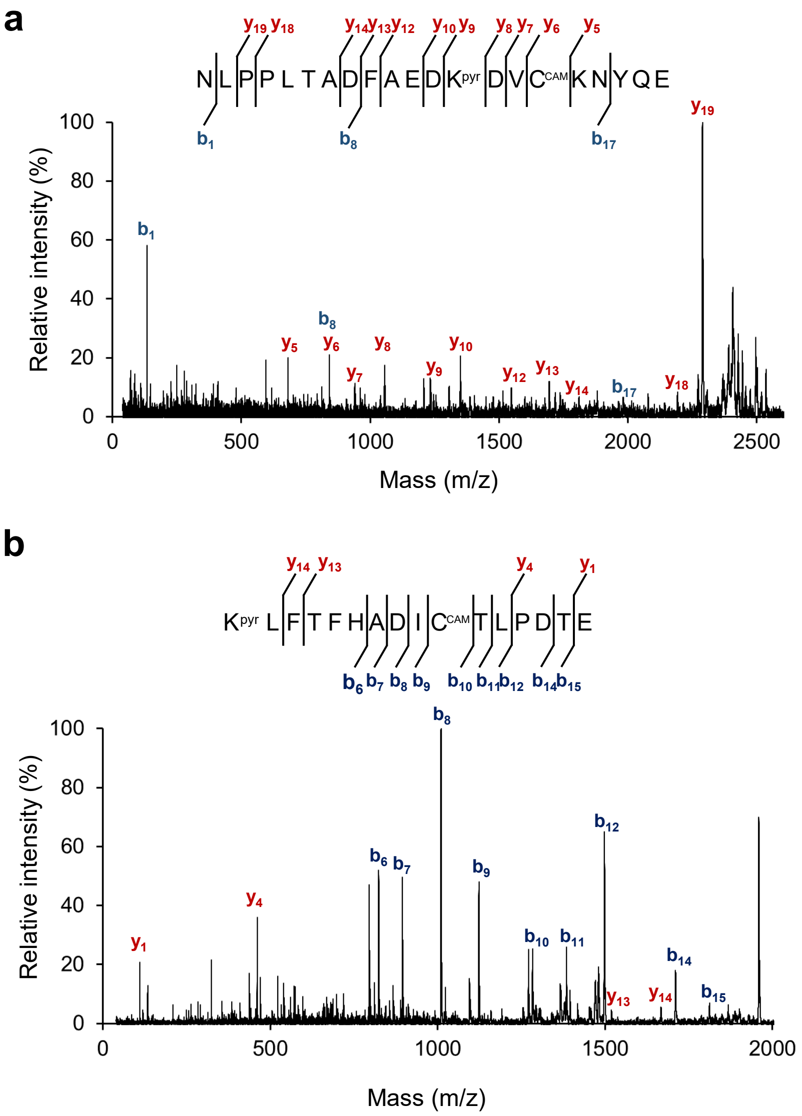

Supplementary Fig. 7. MS/MS spectra of the pyrrolated peptides pyrBSA$_{300\text{-}320}$ and pyrBSA$_{504\text{-}519}$. MS/MS spectra of the pyrrolated peptides pyrBSA$_{300\text{-}320}$ (**a**) and pyrBSA$_{504\text{-}519}$ (**b**). K^{Pyr}, *N*$^{\varepsilon}$-pyrrole-L-lysine; C^{CAM}, *S*-carbamidomethyl cysteine.

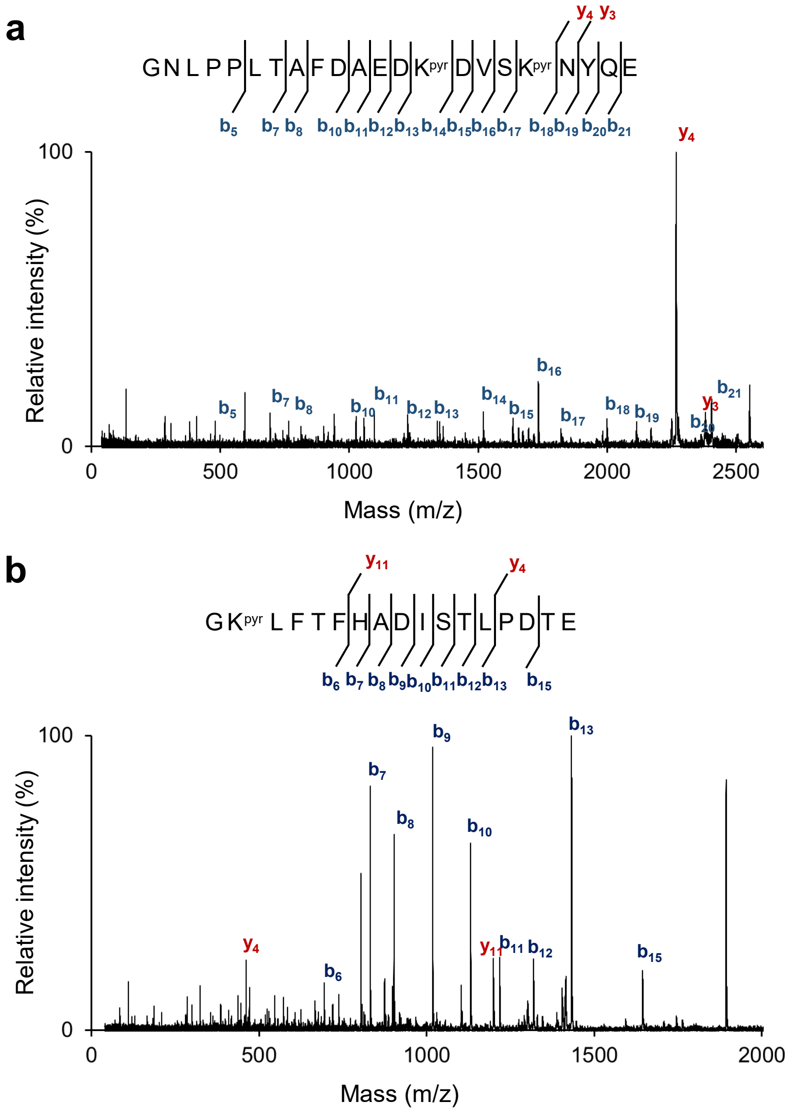

Supplementary Fig. 8. MS/MS spectra of the pyrrolated recombinant peptides pyrBSA$_{300\text{-}320}$ and pyrBSA$_{504\text{-}519}$. MS/MS spectra of the pyrrolated recombinant peptides pyrBSA$_{300\text{-}320}$ (**a**) and pyrBSA$_{504\text{-}519}$ (**b**). K^{Pyr}, N^{ε}-pyrrole-L-lysine.

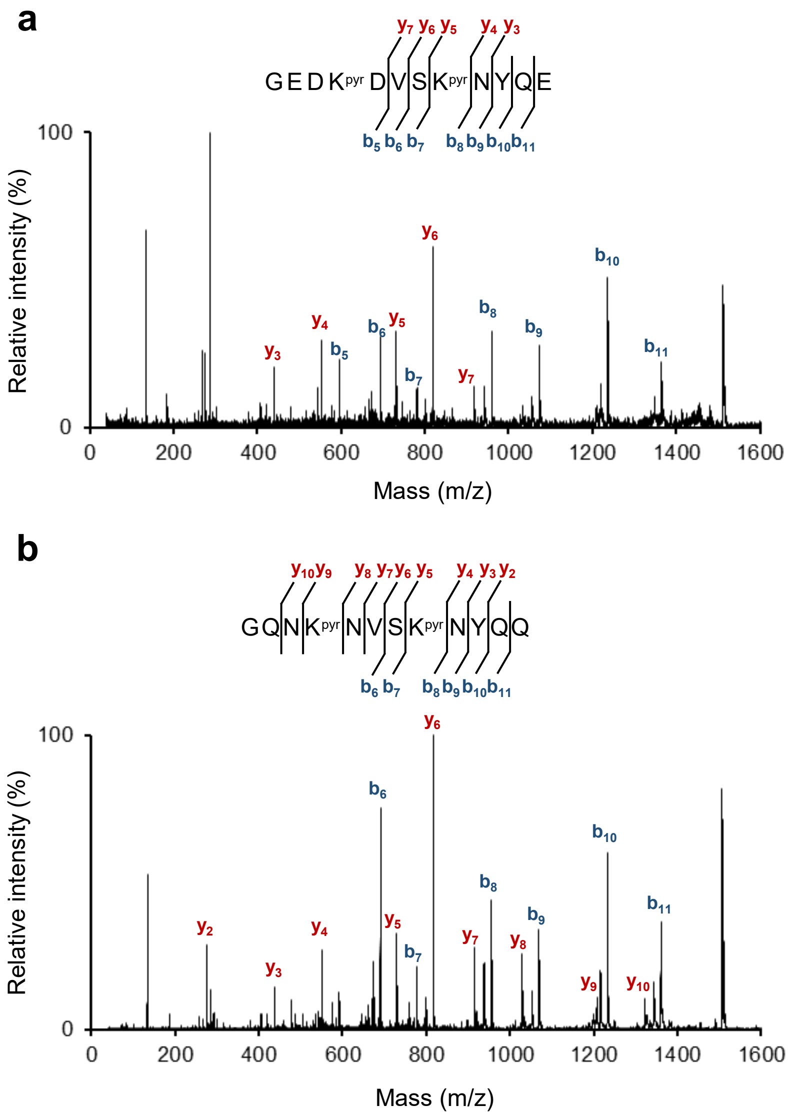

Supplementary Fig. 9. MS/MS spectra of the pyrrolated recombinant peptides pyrBSA$_{300\text{-}320}$ and pyrBSA$_{300\text{-}320}$. MS/MS spectra of the pyrrolated recombinant peptides pyrBSA$_{300\text{-}320}$ (**a**) and pyrBSA$_{504\text{-}519}$ (D>N; E>Q) (**b**); K^{Pyr}, pyrrolelysine.

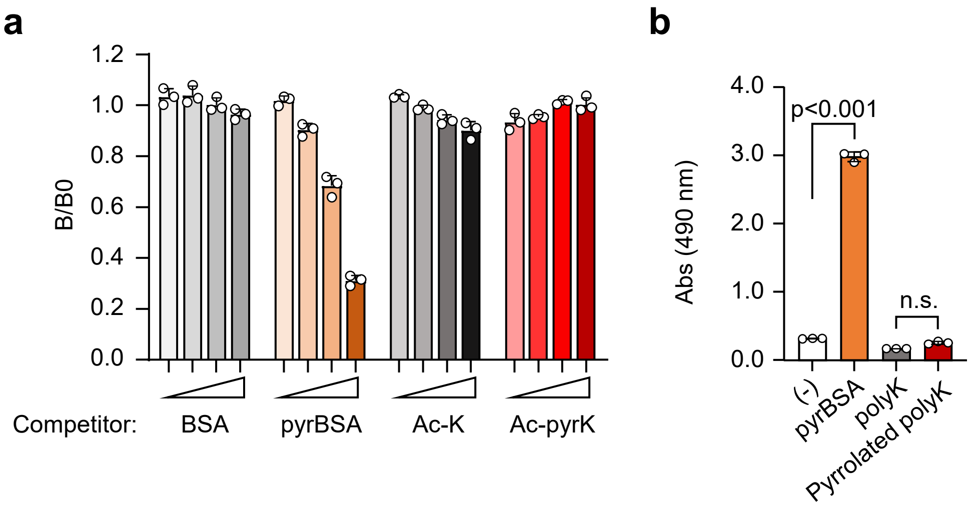

Supplementary Fig. 10. Binding of VH DO2 to pyrK. a, Competitive ELISA using BSA, pyrBSA (6, 30, 150, and 750 nM), Ac-K, and Ac-pyrK (0.8, 4, 20, and 100 μM) as competitors. Ac-K, N^{α}-acetyl-L-lysine; Ac-pyrK, N^{α}-acetyl-N^{ε}-pyrrole-L-lysine. **b**, Binding of VH DO2 to native and pyrrolated polyK. Data are mean ± S.D. of triplicate samples (representative of three independent experiments). Tukey–Kramer test.

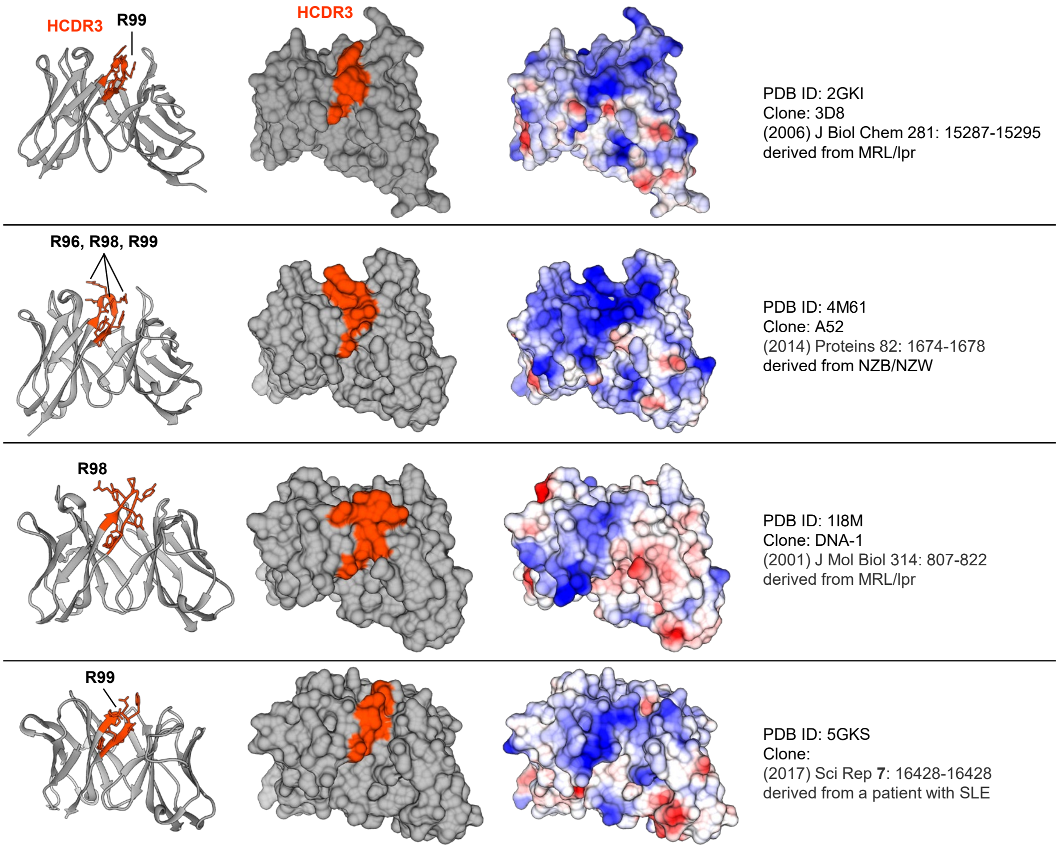

Supplementary Fig. 11. Structural comparison between anti-DNA Abs. Comparison of structures of anti-DNA Abs from SLE mice (3D8, A52, and DNA-1) and SLE patient. Ribon models with HCDR3 highlighted in orange (*left*), 3D surface models with HCDR3 highlighted in orange (*middle*), and with the electrostatic surface potential (*right*) are shown.

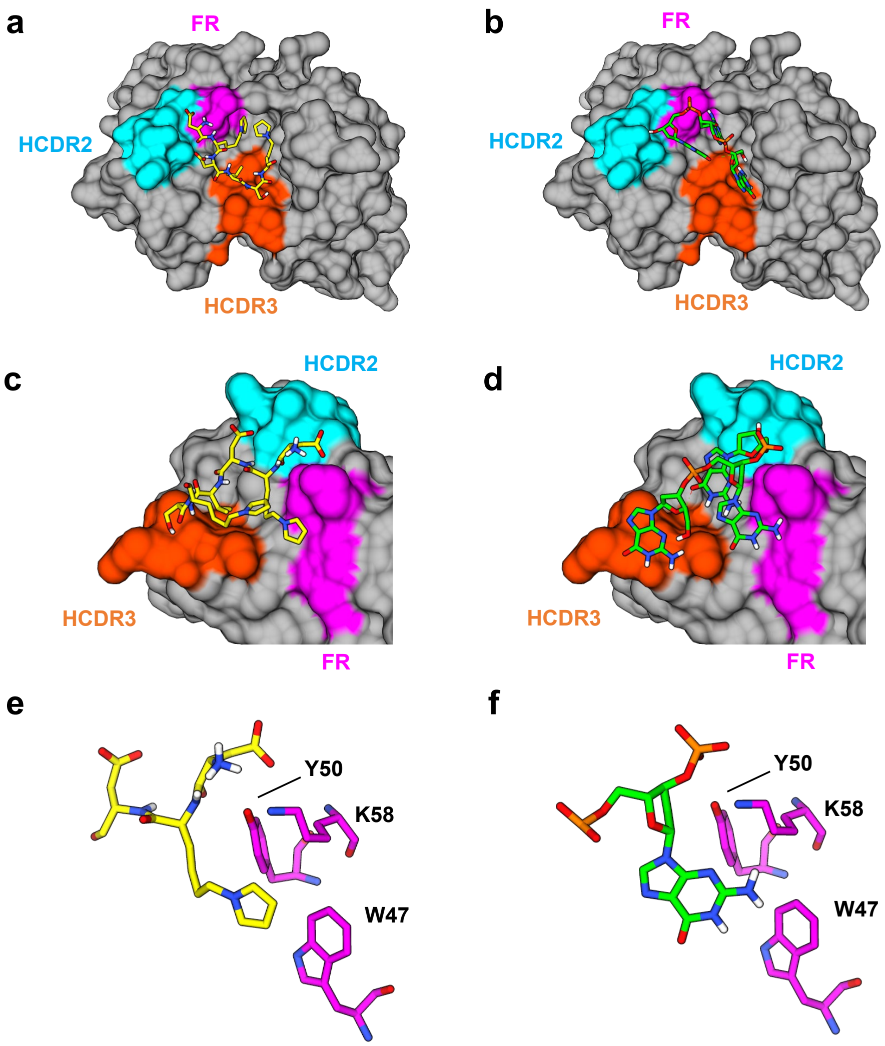

Supplementary Fig. 12. Docking simulation of DO1-clasp with pyrrolated peptides and DNA. Binding models of DO1-clasp with the pyrrolated peptide (**a**, **c**, and **e**) or DNA (**b**, **d**, and **f**) are shown. Pyrrolated peptides and DNA mainly interacted with the VH domains of DO1 clasp (**a** and **b**), especially with HCDR2, HCDR3, and FR around HCDR2 (**c** and **d**). The DO1-clasp is shown as a 3D surface model, and HCDR2, HCDR3, and FR around HCDR2 are colored in cyan, orange, and purple, respectively. W47, Y50, and K58 residues interacted with the pyrrolated peptide (**e**) and DNA (**f**). The pyrrolated peptide (*yellow*), DNA (*green*), and amino acid residues on the FR (*purple*) of DO1-clasp are shown as stick models in the CPK color scheme.

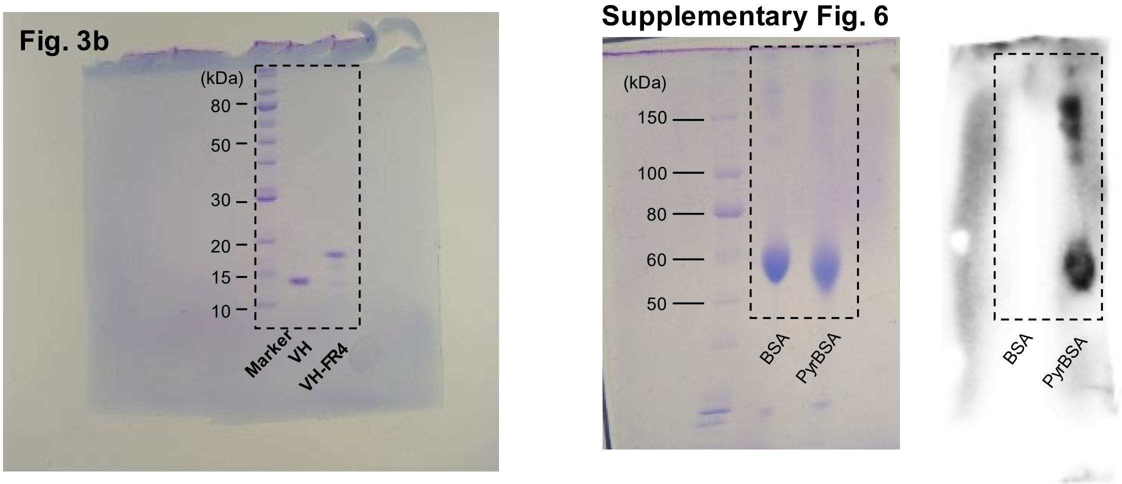

Supplementary Fig. 13. Uncropped gels and blot.
