# Supplementary material for: Molecular and structural basis of anti-DNA antibody specificity for pyrrolated proteins
Source: Commun Biol. 2024 Feb 3;7:149. doi: 10.1038/s42003-024-05851-0 (PMC10838295; doi:10.1038/s42003-024-05851-0)
Supplement: Supplementary file 3 — Description of Additional Supplementary Files [file 42003_2024_5851_MOESM3_ESM.pdf]

Description of Additional Supplementary Files

File name: Supplementary Data 1

Description: Source data for the plots and graphs in the figures.
