# Supplementary material for: Molecular and structural basis of anti-DNA antibody specificity for pyrrolated proteins
Source: Commun Biol. 2024 Feb 3;7:149. doi: 10.1038/s42003-024-05851-0 (PMC10838295; doi:10.1038/s42003-024-05851-0)
Supplement: Supplementary file 5 — Reporting Summary [file 42003_2024_5851_MOESM5_ESM.pdf]

Corresponding author(s): Koji Uchida
Last updated by author(s): Dec 21, 2023

Reporting Summary

Nature Portfolio wishes to improve the reproducibility of the work that we publish. This form provides structure for consistency and transparency in reporting. For further information on Nature Portfolio policies, see our Editorial Policies and the Editorial Policy Checklist.

Statistics

For all statistical analyses, confirm that the following items are present in the figure legend, table legend, main text, or Methods section.

n/a	Confirmed	
☐	☒	The exact sample size (*n*) for each experimental group/condition, given as a discrete number and unit of measurement
☐	☒	A statement on whether measurements were taken from distinct samples or whether the same sample was measured repeatedly
☐	☒	The statistical test(s) used AND whether they are one- or two-sided<br>*Only common tests should be described solely by name; describe more complex techniques in the Methods section.*
☒	☐	A description of all covariates tested
☒	☐	A description of any assumptions or corrections, such as tests of normality and adjustment for multiple comparisons
☐	☒	A full description of the statistical parameters including central tendency (e.g. means) or other basic estimates (e.g. regression coefficient) AND variation (e.g. standard deviation) or associated estimates of uncertainty (e.g. confidence intervals)
☒	☐	For null hypothesis testing, the test statistic (e.g. F, t, r) with confidence intervals, effect sizes, degrees of freedom and P value noted<br>*Give P values as exact values whenever suitable.*
☒	☐	For Bayesian analysis, information on the choice of priors and Markov chain Monte Carlo settings
☒	☐	For hierarchical and complex designs, identification of the appropriate level for tests and full reporting of outcomes
☒	☐	Estimates of effect sizes (e.g. Cohen's d, Pearson's r), indicating how they were calculated

Our web collection on statistics for biologists contains articles on many of the points above.

Software and code

Policy information about availability of computer code

Data collection	The X-ray diffraction data were indexed, integrated, and scaled using the XDS program (XDS-GUI is licensed under the terms of the General Public License Version 2 (GPLv2)). The initial structural models of DO1 were obtained by molecular replacement with MOLREP (Vers 11.9.02) using the coordinates predicted by alphafold2 (AlphaFold (ver.2.3.2)) as the template. Further model building and refinement were performed with REFMAC5 (version 5.8.0352), Coot (version 0.9.8.3), and Phenix (version 1.16-3549). The molecular structures were visualized using UCSF Chimera (version 1.17.1).
Data analysis	Data analysis was performed using GraphPad Prism 6.07. The frame regions and CDRs of the antibody were determined using Antibody Region-specific alignment software (AbRSA). Mass spectrometry analysis was performed by FlexAnalysis 3.4 software (Bruker Daltonics). AutoDock Vina program (version 1.2.0) was used for a docking simulation.

For manuscripts utilizing custom algorithms or software that are central to the research but not yet described in published literature, software must be made available to editors and reviewers. We strongly encourage code deposition in a community repository (e.g. GitHub). See the Nature Portfolio guidelines for submitting code & software for further information.

Data

Policy information about availability of data

All manuscripts must include a data availability statement. This statement should provide the following information, where applicable:

- Accession codes, unique identifiers, or web links for publicly available datasets
- A description of any restrictions on data availability
- For clinical datasets or third party data, please ensure that the statement adheres to our policy

Coordinates and experimental data have been deposited to PDB with accession code 8HYL.

Research involving human participants, their data, or biological material

Policy information about studies with human participants or human data. See also policy information about sex, gender (identity/presentation), and sexual orientation and race, ethnicity and racism.

Reporting on sex and gender	n/a
Reporting on race, ethnicity, or other socially relevant groupings	n/a
Population characteristics	n/a
Recruitment	n/a
Ethics oversight	n/a

Note that full information on the approval of the study protocol must also be provided in the manuscript.

Field-specific reporting

Please select the one below that is the best fit for your research. If you are not sure, read the appropriate sections before making your selection.

☒ Life sciences ☐ Behavioural & social sciences ☐ Ecological, evolutionary & environmental sciences

For a reference copy of the document with all sections, see nature.com/documents/nr-reporting-summary-flat.pdf

Life sciences study design

All studies must disclose on these points even when the disclosure is negative.

Sample size	We did not use statistical methods to determine sample size. We determined the sample size based on similar experimental setups in previous publications.
Data exclusions	No data were excluded.
Replication	Samples were replicated in triplicates and data are representative of three independent experiment.
Randomization	No randomization was used.
Blinding	Investigators were not blinded during data acquisition. Measurements and data reported did not require subjective judgement or interpretation from the investigators.

Reporting for specific materials, systems and methods

We require information from authors about some types of materials, experimental systems and methods used in many studies. Here, indicate whether each material, system or method listed is relevant to your study. If you are not sure if a list item applies to your research, read the appropriate section before selecting a response.

Materials & experimental systems

n/a	Involved in the study
☐	☒ Antibodies
☒	☐ Eukaryotic cell lines
☒	☐ Palaeontology and archaeology
☐	☒ Animals and other organisms
☒	☐ Clinical data
☒	☐ Dual use research of concern
☒	☐ Plants

Methods

n/a	Involved in the study
☒	☐ ChIP-seq
☒	☐ Flow cytometry
☒	☐ MRI-based neuroimaging

Antibodies

Antibodies used

Anti-mouse IgG, HRP-linked Antibody (Cell Signaling Technology, 7076)
Anti P III (g3p) - antibody, monoclonal mouse (MoBiTec GmbH, PSKAN3, clone:10C3)
M13 Major Coat Protein Antibody (RL-ph1) HRP (Santa Cruz Biotechnology, sc-53004 HRP, clone:RL-ph1)
Anti-His-tag mAb-HRP-DirecT (MBL, D291-7, clone:OGHis)

Validation

All antibodies were obtained from commercial sources.

Anti-mouse IgG, HRP-linked Antibody (Cell Signaling Technology, 7076)
Application validated by manufacturer: WB
Validation statements on the manufacturer's website: This product is thoroughly validated with CST primary antibodies and will work optimally with the CST western immunoblotting protocol, ensuring accurate and reproducible results.
Datasheet: https://www.cellsignal.jp/datasheet.jsp?productId=7076&images=1&size=A4

Anti P III D(g3p) - antibody, monoclonal mouse (MoBiTec GmbH, PSKAN3, clone:10C3)
Application validated by manufacturer: WB, ELISA
Validation statements on the manufacturer's website: Every lot is tested for specific binding to antigen in ELISA and Western Blot.
Datasheet: https://www.mobitec.com/media/datasheets/mobitecgmbh/PSKAN3.pdf

M13 Major Coat Protein Antibody (RL-ph1) HRP (Santa Cruz Biotechnology, sc-53004 HRP, clone:RL-ph1)
Application validated by manufacturer: WB, ELISA, IP, IF, and FCM
Datasheet: https://datasheets.scbt.com/sc-53004.pdf

Anti-His-tag mAb-HRP-DirecT (MBL, D291-7, clone:OGHis)
Application validated by manufacturer: WB, ELISA
Datesheet: https://ruo.mbl.co.jp/bio/dtl/dtlfiles/D291-7-ver7.pdf

Animals and other research organisms

Policy information about studies involving animals; ARRIVE guidelines recommended for reporting animal research, and Sex and Gender in Research

Laboratory animals

21-week-old MRL/lpr mice (MRL/MpJJmsSlc-lpr/lpr) were used in this study.

Wild animals

No wild animals have been used for this study.

Reporting on sex

We used female MRL/lpr mice only because SLE is more frequent in females.

Field-collected samples

No field collected samples have been used for this study.

Ethics oversight

All experimental procedures were conducted following protocols approved by the Institutional Animal Care and Use Committee at the University of Tokyo (Permission No. P21-025).

Note that full information on the approval of the study protocol must also be provided in the manuscript.
